# Outcomes that matter: A qualitative study with persons with schizophrenia and their primary caregivers in India

**DOI:** 10.1016/j.ajp.2012.06.002

**Published:** 2012-09

**Authors:** Madhumitha Balaji, Sudipto Chatterjee, Beth Brennan, Thara Rangaswamy, Graham Thornicroft, Vikram Patel

**Affiliations:** a Sangath Centre, 841/1 Alto-Porvorim, Bardez, Goa 403521, India; b International Health Division, Abt Associates, Inc., 4550 Montgomery Ave, Bethesda, MD 20814, United States; c Schizophrenia Research Foundation Centre, R/7A, North Main Road, Anna Nagar West Extension, Chennai 600101, India; d Health Service and Population Research Department, Institute of Psychiatry, King's College London, De Crespigny Park, London SE5 8AF, United Kingdom; e London School of Hygiene and Tropical Medicine, Keppel Street, London WC1E 7H, United Kingdom

**Keywords:** Schizophrenia, In-depth interviews, Low and middle income countries, India, Thematic analysis, Content analysis

## Abstract

**Background:**

Involving persons with schizophrenia and their families in designing, implementing and evaluating mental health services is increasingly emphasised. However, there is little information on desired outcomes from the perspectives of these stakeholders from low and middle income countries (LMIC).

**Aims:**

To explore and define outcomes desired by persons with schizophrenia and their primary caregivers from their perspectives.

**Method:**

In-depth interviews were held with 32 persons with schizophrenia and 38 primary caregivers presenting for care at one rural and one semi-urban site in India. Participants were asked what changes they desired in the lives of persons affected by the illness and benefits they expected from treatment. Data was analysed using thematic and content analysis.

**Results:**

Eleven outcomes were desired by both groups: symptom control; employment/education; social functioning; activity; fulfilment of duties and responsibilities; independent functioning; cognitive ability; management without medication; reduced side-effects; self-care; and self-determination. Social functioning, employment/education and activity were the most important outcomes for both groups; symptom control and cognitive ability were more important to persons with schizophrenia while independent functioning and fulfilment of duties were more important to caregivers.

**Conclusions:**

Interventions for schizophrenia in India should target both clinical and functional outcomes, addressing the priorities of both affected persons and their caregivers. Their effectiveness needs to be evaluated independently from both perspectives.

## Highlights

► Eleven outcomes spanning clinical and functional domains are important to persons with schizophrenia and primary caregivers. ► Symptom control is a very important outcome for persons with schizophrenia. ► Social functioning, employment and activities of daily living are prioritised outcomes for both groups. ► Interventions for schizophrenia in India need to target multiple outcomes.

## 1 Introduction

The emergence of the recovery paradigm (Roe, 2001; Liberman et al., 2002) has brought into focus the need for incorporating the views and aspirations of persons with schizophrenia (PwS) in designing, implementing and evaluating mental health care services (Robert et al., 2009). Subjective experiences of the illness and the needs of PwS can affect adherence to interventions and perceptions of care received (Fenton et al., 1997; Kikkert et al., 2006; Pyne et al., 2006). As families are closely involved in care giving and are considerably affected by the illness (Thara et al., 2003a; Jagannathan et al., 2011), their perspectives are also important. Their involvement may result in better outcomes for PwS and enhance their engagement in and satisfaction with health services (Falloon et al., 1985; Dixon and Lehman, 1995; Chue, 2006). This is particularly true in countries like India, where PwS typically live with their families, and the latter often participate in decision-making regarding health care and treatment compliance (Srinivasan and Thara, 2002; Chatterjee et al., 2009).

Outcome priorities for schizophrenia have been generated from service-user perspectives and that of families and other stakeholders (Fischer et al., 2002; Cradock et al., 2002; Shumway et al., 2003; Rosenheck et al., 2005; Ng et al., 2008, 2011). Desired outcomes generally include symptom remission, reduction of side-effects, employment, independent living, remission without medication, and improved relationships. Studies comparing outcome priorities of individuals and families, however, show conflicting results. One study, for example, shows that both groups agree on outcomes that are important, such as relationships and independence (Cradock et al., 2002), while another demonstrates that they differ in their priorities, with families placing a greater emphasis on social relationships and housing independence and PwS, on control of side-effects and work performance (Fischer et al., 2002). Moreover, most of the available literature on desired outcomes comes from high resource countries. This gap in information is a potential barrier to designing and evaluating contextually appropriate services for PwS and their families in low and middle income countries (LMIC).

This paper describes a qualitative study conducted in India which sought to explore and define outcomes in schizophrenia desired by PwS and their primary caregivers. This study was part of the formative phase of a randomised controlled trial designed to evaluate the effectiveness of a community based intervention for schizophrenia in India (Chatterjee et al., 2011).

## 2 Method

### 2.1 Participants

We used purposive sampling and selected two sites for our study, one rural and one semi-urban, to maximise the richness and variety of data on desired outcomes. The semi-urban site was Goa (nearly 50% of the population are urban), a small state on the west coast with a population of about 1.4 million. The main sources of employment here include tourism and agriculture. Over 80% of people are literate. The rural site comprised three blocks in the Kancheepuram district of Tamil Nadu (TN) state in south India, where the main source of employment is agriculture and the combined population is over 700,000. Literacy is over 70%. Within each site, we recruited two groups of participants, PwS and primary caregivers, from those presenting for treatment at community mental health clinics (rural site) or psychiatric treatment facilities (semi-urban site) on a first come-first serve basis. PwS were eligible to participate if they met the ICD-10 criteria for schizophrenia and were equal to or above 18 years of age. Psychiatrists in these centres made the diagnosis. The primary caregiver was identified by the PwS and accompanying family members or psychiatrists as the person in the family primarily responsible for meeting emotional, financial and health needs of the PwS.

### 2.2 Data collection

In-depth Interviews (IDI) were then conducted with PwS and caregivers between September 2008 and July 2009. Participants were asked about their perceptions of the illness and experiences of care received *(What do you think you suffer from? How has the illness affected your life? What benefits have you experienced from treatment?*). Responses to these questions then served as useful probes for eliciting information on desired outcomes in the form of changes they wanted in the lives of the PwS and the benefits they expected from treatment (*Can you tell me what changes you want in your life? What benefits do you seek from treatment?*). Follow-up questions were largely based on what the participants said in response to these open-ended questions and varied from interview to interview: *What sort of changes do you want in* _______ *[outcome mentioned by participant]?* or *(for changes in specific outcomes mentioned) For which [symptom/side-effect/relationship]?* Persons who did not respond adequately were probed in a modified manner, for example by drawing their attention specifically to areas of impact previously mentioned and probing about whether and in what manner changes were desired in these areas, for example: *You said that you are having* ____________ *[problem mentioned earlier by participant] how would you define getting better? What changes do you wish to make? For example, do you want your symptoms to change? Which ones? In what way?* We ensured that the probes used were tailored to the specific interview and were in keeping with the established guidelines through rigorous supervision. The interview guides can be found on our website www.sangath.com.

Two research assistants (RAs) at each site conducted the interviews. Before this, they participated in an intensive 3-day workshop on qualitative interviewing methods and underwent subsequent training for 30 days on the use of the guides. Training included lectures, video tapes of interviews, practice sessions with PwS and caregivers, and role plays observed by trainers. Interview guides were translated into local languages (Konkani in Goa and Tamil in TN) by the RAs. They were revised at midpoint taking into consideration researcher experiences and findings from interviews with 12 PwS and 16 caregivers (included in the sample for analysis). The number of questions was reduced, especially in the PwS guide, to reduce burden on participants; there were fewer probes, allowing for more open-ended probing; and words in the local languages that participants did not understand were substituted.

### 2.3 Procedure

Assent for participation in the study was obtained by the psychiatrists. Those who assented were given informed consent. Written consent was obtained from literate persons and verbal consent was tape recorded for those not literate. Participants who consented were then interviewed either at their homes or at the treatment facility. Each interview was tape recorded and took approximately 45 min. Interviews with PwS and caregivers who were from the same family were carried out simultaneously, in separate, privately enclosed spaces by two independent RAs. Guidelines for transcription and translation were standardised for both sites. Interviews were transcribed and then translated into English within a week. Field notes were stored in a locked cabinet. Audio files and transcriptions were stored in computers and password protected to restrict access to authorised team members.

### 2.4 Data analysis

All conducted interviews were analysed in NVivo 8 using thematic analysis and content analysis (Miles and Huberman, 1994; Braun and Clarke, 2006; Namey et al., 2007). In the first stage, a coding framework was developed that was based on the research question. This consisted of “master codes” (i.e., abbreviations for the main categories of data expected to emerge from analysis). Examples of master codes were “[o]” for the variable “outcomes”; and “[p]” for PwS or “[c]” for caregivers, to denote respondent group perspectives. The codes in the framework were minimal in order to maximise inductive generation of themes.

In the next stage, raw data was read and re-read repeatedly in order for the coders to become familiar with and get immersed in data. It was then broken down into and summarised as smaller fragments of meaningful information (codes). These were at first descriptive (i.e., paraphrases of words used by the participants themselves) and then interpretative (i.e., words chosen by the coders as more representative of the ‘underlying meanings’ in data). Codes that were similar to one another (reflecting the same meaning) were then grouped together as one category. For example, the desire for doing housework without help was grouped together with other desires of working and earning, and going to the market by oneself, on the key aspect of independence. Next, the category was given a label in the English language that was the closest available approximation to the meaning of the category, and that would most succinctly capture the nuances of the category in the local context (outcome domain). In the case of the above example, this was “independent functioning”. Lastly a ‘definition’ was applied to each category label which comprised of an explanatory statement that united its individual codes on consistency and meaning. For example, the definition of “*taking care of personal needs without being dependent on or having the help/assistance of others*” was given to “independent functioning”. In some cases, the same codes were categorised in multiple outcome domains; for example, doing housework was categorised under both domain “activity” and domain “independent functioning” if it was important for the participant that these tasks be done by PwS without being assisted. Patterns were then derived from across-case analysis and involved comparing and contrasting emergent themes between classes of participants (e.g., men and women). Observed differences are highlighted in Section 3.2.

We then did a content analysis of the outcome domains in order to understand their relative importance for each respondent group. This was based on two factors: (i) *saliency (s)* of the outcome, i.e., the total number of references to that outcome in transcripts and (ii) *frequency (f)* of the outcome, i.e., the number of participants who wanted that outcome. The s values in transcripts which were outliers (i.e., exceeded the average *s* for that outcome by at least two times) were replaced with the average value, to minimise overestimation of the salience of that outcome.

The first author coded all interviews. Two other persons, including another author (BB), independently coded 15 and 18 randomly selected interviews to check the meanings of the codes and the consistency of findings. Once the analysis was completed, outcome domains were compared to ensure that they meant the same to all coders. Areas of disagreement were resolved through discussion and consensus was reached (Mishler, 1986).

### 2.5 Ethical approval

Institutional Review Boards (IRBs) at Sangath and Schizophrenia Research Foundation Centre approved the study.

## 3 Results

### 3.1 Sample

Thirty-two PwS and 38 caregivers took part in this study (Tables 1a and 1b). The mean age of PwS was 42 years and the gender ratio was roughly even (53% female participants). Fourty-four percent were married and 31% had completed high school. 72% were not employed. Caregivers included parents (44%), siblings (13%), children (11%), spouses (21%), and family by marriage (11%). They tended to be older than PwS and were mostly female (63%) and married (76%). Twenty-seven percent had completed high school.

### 3.2 Defining desired outcomes

Eleven outcomes were identified as being desired by both respondent groups. These were symptom control; activity; employment/education; social functioning; fulfilment of duties and responsibilities; independent functioning; cognitive ability; management without medication; reduction of side-effects; self-care; and self-determination (described below).

#### 3.2.1 Symptom control

Both PwS and caregivers wished for reductions in or elimination of what they perceived to be health experiences characteristic of the illness. PwS desired reductions in symptoms of thought and perception (e.g., hearing of voices or “negative” thoughts), changes in mood (e.g., being “eager”, not having “fear”) and wanted to be “healthy”. About two-thirds were concerned with somatic complaints (e.g., aches and pains). Some feared that previously experienced symptoms (“stroke” or “phases”) would return. Caregivers, in addition to the above, wished for control of behaviours (e.g., anger or physical abuse, wandering or inappropriate laughter). Caregivers of male PwS (13/17) reported this more than caregivers of female PwS (10/21).

Voices in my ears should stop. That is troubling me, even after taking tablets. (PwS, 45 years, female, TN)

I told the doctor that my neck, legs and hands were aching and that I am not able to breathe properly. If they give an injection my pain would go and my illness will go. (PwS, 65 years, male, TN)

I am frightened of whether he (PwS) will abuse me in the future. He should not make us feel ashamed. He goes out and eats something or the other; he begs and grabs food from the children… if he is not like that, it is enough. (Caregiver, mother, 75 years, TN)

#### 3.2.2 Activity

Activity referred to increases in levels of movement and mobility of PwS and their engagement in productive tasks of daily living. This included being “energetic”, “quick”, and “moving about” (e.g., not being lazy or sleeping “all the time”); following a routine (e.g., waking up/eating on time); exercising and being “fit”; keeping busy (e.g., pursuing hobbies such as reading or making handicrafts); and/or being engaged in some form of productive tasks (e.g., cooking, cleaning or other housework) and performing these satisfactorily. This outcome was more frequently reported by urban caregivers (13/18) compared to rural (12/20). It was only reported by women with schizophrenia and was more frequently reported by caregivers of women (17/21) than men (8/17).

(I need to know) how to (keep to) timings. I get up very late, I feel lazy. I need to have proper food at proper times. (PwS, 46 years, female, Goa)

She (PwS) just sits at home the whole day. She has to do some work… like earlier she would be praying to god, doing prayer rituals, cleaning the fish, and so on. She has to read books; first she used to read but now she is not reading. I want her to play like the way she used to play before… she was always good in everything – singing, playing the tabla (musical instrument), playing cricket. (Caregiver, mother, 74 years, Goa)

#### 3.2.3 Employment/education

Employment was defined as PwS “working” or “having a job”. This included seeking work or resuming previously held jobs, attending work regularly, or/and performing work with competence and efficiency. Education was defined as PwS “studying”, for example, completing high school or university or pursuing higher degrees; this was mainly sought for younger persons, all of whom were urban, and who had dropped out of school or had discontinued their studies as a result of the illness. Men with schizophrenia (9/15) reported this outcome more frequently than women (5/17) as did caregivers of men (14/17), compared to those of women (8/21).

I used to go to college… now I’m not going to college, I’m sitting at home. And all my friends are well, going to college. I should start attending my classes. I should start studying as I used to study before. I want to complete engineering in four to five years. (PwS, 23 years, male, Goa)

I say to him (PwS) “Please obey my words, we need to look after the family… think of that and do your work properly”. He has to go for work; only then he can be happy with his earnings. When he earns he can spend… he will be respected more… if he does not depend on his mother and father. (Caregiver, mother, 65 years, TN)

#### 3.2.4 Social functioning

Social functioning referred to PwS socialising and having meaningful interpersonal relationships. Participants wanted PwS to be able to have social skills (have manners, for e.g., greeting others); “go out” and meet people more often; have friends to “talk to” or spend time with; attend and participate in family and social functions; get married; and/or be reunited with or repair broken relationships with spouses and family members. This was a little more frequently reported by caregivers of women (13/21) than men (7/17).

Now I don’t require the tablet treatment, I require training. Meaning how to co-operate with others, change the way I speak and how to make friendship… I had control over all my friends. Now they have all gone away, so I want that situation to be all right now. (PwS, 19 years, female, Goa)

He (PwS) has to move about in society… finding a life partner. Someone who will understand him; if he finds a life partner, I will feel that half my responsibility is over. (Caregiver, mother, 52 years, Goa)

I thought she (PwS's wife) is my daughter-in-law, we will go and bring her back; suppose I die, what will be my son's state?!… When he is going to reunite with his family, I don’t know! Only on the day that it happens I will be happy! (Caregiver, mother, 63 years, TN)

#### 3.2.5 Fulfillment of duties and responsibilities

This outcome referred to PwS performing duties and responsibilities seen as befitting their age, gender and role within the family or society. The type of duties expected varied from case to case, but in general included working, earning and supporting family members; representing family at social gatherings such as weddings; and looking after the house and its belongings. For example, PwS who were parents were expected to be (or expected themselves to be) “good” mothers or fathers by providing for children financially and arranging their marriages, and PwS who were sons or daughters, to be independent, “loving”, “obey” their parents, and “cook food” for them. Caregivers generally expected PwS to “do work at home”, “take care of children” and “attend social gatherings” but these were more commonly expected from women in this group whereas men were expected to earn and support their family. This outcome was more commonly reported by rural participants (16/20 caregivers and 6/15 PwS) than urban participants (7/18 caregivers and 1/17 PwS).

He (PwS) must go for work and should support me. If he earns I will think that he is there to take care of my house. (Caregiver, mother, 61 years, TN)

She (PwS) should be like how she was when we were small children… she took take care of us well… now she eats and she sleeps, she should not be lazy, I want her to be responsible at home. She (PwS) is not clear about who it is she is talking to, she doesn’t know what to talk. If our marriage is fixed… they (in-laws) shouldn’t say that our mother is mad, that we are the daughters of a mad person. So she should become well, only then some one will marry us, and our life can change. (Caregiver, daughter, 17 years, TN)

I tell (PwS) “Finish your internship, take up a job as a medical officer… you can decide your future, your parents cannot decide your future”. Children after completing studies should stand on their own legs and start earning. (Caregiver, father, 60 years, Goa)

I am the only one in the family who is not working, who is not helping (them)… till now my brother did lot for my family… I feel like I’m the person who should do something for my family (now)… it is the age of my brother to get married… because of my health, I am not able to help him. (PwS, 23 years, male, Goa)

#### 3.2.6 Independent functioning

Independent functioning was defined as the ability of the PwS to take care of their personal needs without being dependent on or having the help/assistance of others. This was desired in several areas – doing daily tasks for which the person was responsible without being assisted (e.g., household chores such as cooking, cleaning); earning an income and meetings one's own expenses of food or clothing without being dependent on the earnings of others; and travelling to places (e.g., the market or to relatives’ houses) without being accompanied by others. Only one caregiver mentioned staying (temporarily) away from family. Caregivers of women (13/21) reported it slightly more frequently than caregivers of men (7/17).

I want to do the work without anyone's help. When somebody comes home I expect them to help me with my housework. I wish to live independently… go to the shop … now I am scared, now I am not able to go out on my own. (PwS, 45 years, female, Goa)

If she (PwS) works and she earns, then she can keep the money for her expenses… she will not expect us to provide for her. She can buy jackets, she can buy and wear saris (Indian garment)… she can be how she wishes to be. (Caregiver, sister-in-law, 40 years, TN)

#### 3.2.7 Cognitive ability

Cognitive ability referred to improvement in functions of cognition related to orientation, memory, and concentration. This meant PwS being less “confused”; being able to remember things and being less forgetful (e.g., with respect to their day to day tasks); being able to concentrate on or pay attention to their work, household tasks or to conversations with other people; and being “alert”, with a “sharp” mind. It was more common amongst urban PwS (6/17) than rural (2/15). In the caregiver sample, all persons who reported this were caregivers of women.

I have problems in memorizing phone numbers. I was able to recollect my phone number only after two years. I should remember things, I cannot memorise. (PwS, 50 years, male, Goa)

#### 3.2.8 Self-care

Self-care was defined as PwS taking care of their own health and maintaining good hygiene practices. Examples included taking medicines on their own without being persuaded or reminded to; informing others when feeling unwell; sleeping well and having healthy eating habits (e.g., having a good appetite and eating proper meals); bathing regularly and being clean; taking care of personal and household belongings; not smoking cigarettes or inhaling tobacco; and maintaining a neat and groomed appearance by combing one's hair and dressing appropriately. Rural PwS and caregivers tended to report this more frequently (12/20 and 5/15 respectively) than urban participants (6/18 and 1/17 respectively).

She (PwS) is very unhygienic… so we don’t like to eat something made by her. She never even washes the tea cup in which she drinks tea. She has to be clean… I made her take a bath and change the dress and clean the clothes. (Caregiver, daughter, 21 years, TN)

She (PwS) needs to take tablets on her own. She must not expect someone to give her tablets. One day we may not be able to give them to her, another day we may not find time to do so. (Caregiver, sister-in-law, 38 years, TN)

#### 3.2.9 Management without medication

Participants wanted PwS to “be all right” without having to taking medication. This was reported by PwS from both the urban and rural sites but only by urban caregivers.

I don’t want to really totally on tablets. Because I want to be normal again without tablets. (PwS, 23 years, male, Goa)

I would be very happy if this medication would have been cut off slowly slowly… that's always in my mind – how long is she going to take the side-effects! Is it necessary she has to take it (medication)? (Caregiver, mother, 61 years, Goa)

#### 3.2.10 Reduced side-effects

Both groups wanted side effects of medication such as tremors or weight gain, to stop.

I should be good and the medicines should be reduced. I put on weight. I immediately asked the doctor, “Are they that make me put on weight!?” (PwS, 63 years, female, Goa)

#### 3.2.11 Self-determination

Self-determination referred to changes in negative attitudes or personality traits of PwS and their acquiring coping strategies to deal with problems. This included having confidence in oneself; being “bold” and facing challenges (e.g., going to social gatherings and facing people); and being accomplished in or having the desire to excel in one's profession (e.g., aspiring to be an artist). Caregivers especially wanted PwS to listen to and accept their advice or offers of help; to be less miserly or selfish; to be able to forget or recover from traumatic events in their past; to have insight into the consequences of one's actions (e.g., embarrassment for the family) and to think positively about one's future.

I want that thing to be thrown away – that I look ugly. My mind should feel that I look beautiful. What I want is for these negative and irrational feelings to be thrown away from me. (PwS, 29 years, female, Goa)

(PwS has to be) more open minded… to give up dominating (me). She cannot be thinking of herself; if she has to live in society she has to think of the needs of others. She's not ready to let go of all that has been done to her (husband's physical abuse). I know it's not easy when somebody has been bad to you, but that person is dead and gone, he's not alive anymore to do anymore harm to you. So let it go! (Caregiver, daughter-in-law, 34 years, Goa)

### 3.3 Relative importance of outcomes

Table 2 shows the relative importance of desired outcomes for PwS and caregivers. From this table it can be inferred that the outcome likely to be the most important for PwS is symptom control, reported by over half the participants (frequency *f* = 17, saliency *s* = 37), followed by employment/education (*f* = 14, *s* = 28) and social functioning (*f* = 14, *s* = 16). Others that seem to be important, but relatively less so (reported by less than one-third of PwS) are activity, cognitive ability, management without medication, fulfilment of duties and responsibilities, reduced side-effects, independent functioning, self-care, and self-determination. Similarly, the most prioritised outcomes for caregivers can be judged to be activity (*f* = 25, *s* = 53), employment/education (*f* = 22 *s* = 51), fulfilment of duties and responsibilities (*f* = 23, *s* = 47), social functioning (*f* = 21 *s* = 45), symptom control (*f* = 23, *s* = 35) and independent functioning (*f* = 20, *s* = 35). Outcomes of less importance are self-care, self-determination, reduced side-effects, management without medication, and cognitive ability. Findings are similar for both groups; employment/education, activity and social functioning, for example are in the top four of both lists. However there seem to be differences with respect to the relative importance of some of the others; symptom control and cognitive ability are likely to be more important to PwS, and independent functioning, fulfilment of duties and responsibilities and self-care to be more important to caregivers.

## 4 Discussion

This study explores the desired outcomes for schizophrenia, from the perspectives of PwS and primary caregivers, in rural and urban populations in India. We identified 11 outcomes which we propose represent the relevant domains to recovery from this serious mental illness in the Indian context (Table 3). Three of these, symptom control, reduced side-effects, and cognitive ability reflect domains of clinical recovery while the remaining eight, namely activity, social functioning, education/employment, independent functioning, fulfilment of duties and responsibilities, self-care, management without medication and self-determination, constitute domains of functional recovery.

Although the relative importance of outcomes was similar for both PwS and caregivers, in particular, for social functioning and employment/education, differences were observed with respect to some of the other outcomes such as symptom control, fulfilment of duties, and self-care. These differences may result in the two groups having divergent views about the goals of interventions, affecting their involvement in and satisfaction with health services. Given that most PwS live with their caregivers, and the latter play an important role in providing emotional support and ensuring treatment compliance, interventions may therefore need to be flexible to also address the outcomes that they prioritise. The generation of treatment goals should be a collaborative effort, emphasising the gathering of relevant information and assessment of needs from both parties. Similarly, evaluations of treatments need to incorporate both perspectives, using appropriate PwS and caregiver reported outcome measures (McCabe et al., 2007). The outcome of management without medication considered important by both PwS and caregivers may clash with care providers’ views of the necessity of medication. This can act as a potential barrier to the formation of therapeutic alliances and to PwS’ adherence to treatment (Ng et al., 2008, 2011). The role of the health care provider is, thus, crucial to initiating collaboration of all parties in treatment, facilitating discussions, revisiting differences, and setting mutual goals for interventions through the process of psycho-education.

Outcomes such as social functioning, activity and employment (in the top four of both lists) are contextually relevant to India. Disabilities in these domains are associated with increased risk of relapse (Rajkumar and Thara, 1989). These can also severely affect quality of lives of PwS and their families. Separation or divorce for example, followed by women moving back into parental homes and being dependent on families for financial support results in hostility and lack of acceptance by family members (Thara et al., 2003b). Unemployment too can cause tremendous strain on families who were previously dependent on PwS for livelihood and who now face considerable financial burdens as a result of the illness (Grover et al., 2005). On the other hand, improvements in these are associated with decreases in symptoms and good marital outcomes (Srinivasan and Thara, 1997a). Our findings also indicate culturally relevant differences within respondent groups. Gender differences were observed; for example, men were more frequently expected to be employed, and women to care for children and do housework. These findings are consistent with gender roles prevalent in India, and have been observed in prior research (Srinivasan and Thara, 1997b, 1999). There were also some rural-urban differences; education was desired by urban participants while fulfilment of duties and responsibilities was more commonly desired by rural participants. Such ‘local flavours’ in desired outcomes indicate the need for interventions to target culturally relevant domains and to develop corresponding parameters for measuring (taking into consideration the meaning of these outcomes in the local context) (Cohen, 1992; Isaac et al., 2007).

We were able to identify four other studies, whose aims were the most comparable with that of ours. One study from China (Ng et al., 2008) held focus groups with eight persons with chronic schizophrenia regarding what constituted recovery. The other studies are from the USA. Cradock et al. (2002) explored stakeholder perspectives through focus groups (31 persons and 14 caregivers) and questionnaires (23 persons and 7 caregivers). Fischer et al. (2002) asked 20 PwS and 20 family members to rank seven outcomes predetermined by expert consensus as being relevant to schizophrenia in order of preference. Rosenheck et al. (2005) asked 1200 PwS to rank 6 outcomes in the order of their importance. Our findings of desired outcomes (notably, such as activity, social functioning and employment) are consistent with those described by these studies, indicating that many outcomes for schizophrenia may be similar across cultures, i.e., ‘universal’. On the other hand, some of the domains reflect a more contextually relevant perspective. Fulfilment of duties and responsibilities, for example, reflects the widely held beliefs in Asian societies as to what constitute culturally appropriate practices within the family (Gorden and Shapiro, 1994). Similarly, though independence was a valued outcome across cultures, living alone was emphasised in the USA, while in our study the emphasis was on being able to work at home, travelling without help, and earning and supporting one's needs, while continuing to live with families. Other contextually appropriate observations related to gender and rural-urban differences have been noted earlier.

There are three limitations in our study. The first is that the relative importance of outcomes (Table 2) was derived from analysis and not from the participants themselves, for example, by asking them to rank the outcomes. However, this is also a strength in that the outcomes were derived from the data (rather than a priori as was the case with some of the other studies). Secondly, it is possible that there were more functional outcomes reported here because our participants were not “treatment naïve”. However, symptom control still emerged as a very important outcome for PwS, perhaps indicating that pharmacological treatment alone (which comprises the care received for most PwS in LMIC) is insufficient in achieving symptom control and that diverse approaches to the management of this condition are important. Pharmacological treatments are, moreover, associated with unpleasant side effects, indicating the need for alternative interventions which may be experienced as being more “pleasant” (Halliburton, 2003). The final limitation is that our study did not allow for comparisons of persons at different stages of the illness, the assumption being that desired outcomes change over time; however this was considered to be beyond the scope of our study design.

To the best of our knowledge, this is one of the few qualitative studies in LMIC which sought to elicit desired outcomes for schizophrenia from PwS and their caregivers. Our results strongly indicate the need for interventions in India to target both clinical and functional outcomes, addressing and evaluating the priorities of PwS and their caregivers, and tailoring the intervention to their requirements. As desired outcomes span a range of needs, interventions will need to use a combination of clinical as well as psychosocial interventions (Mari et al., 2009; Chatterjee et al., 2009). Such an approach has been shown to be acceptable and feasible in India (Chatterjee et al., 2009) and is currently being evaluated for effectiveness in the Community care for People with Schizophrenia in India (COPSI) trial (Chatterjee et al., 2011).

### Role of funding source

This work was supported by the Wellcome Trust, UK. The Wellcome Trust had no other role in the study.

### Contributors

MB helped conceptualise the project, developed the interview guides, did the analysis, interpreted the data and wrote the paper for publication. BB helped develop the interview guides and was involved in the analysis and interpretation of findings. SC, TR, VP and GT designed the project and critically revised the paper.

## Conflict of interest

The authors have no conflict of interest.

## Figures and Tables

**Table 1a:** Socio-demographic characteristics of persons with schizophrenia (PwS).

Socio-demographic variable	Sample (*N* = 32)
Age (in years)	
Mean age	42
Age range	
17–29	7 (22%)
30–39	6 (19%)
40–49	10 (31%)
50 and above	9 (28%)
Gender	
Female	17 (53%)
Male	15 (47%)
Marital status	
Single	10 (31%)
Married	14 (44%)
Divorced/separated	5 (16%)
Widowed	3 (9%)
Education (highest level completed)	
No formal education	2 (6%)
Primary school or lower	4 (13%)
Middle school	16 (50%)
High school	9 (28%)
University	1 (3%)
Occupation	
In employment	7 (22%)
Not in employment	23 (72%)
Currently studying	1 (3%)
Retired	1 (3%)

**Table 1b:** Socio-demographic characteristics of primary caregivers.

Socio-demographic variable	Sample (*N* = 38)
Age (in years)	
Mean age	50
Age range	
17–29	5 (13%)
30–39	5 (13%)
40–49	6 (16%)
50 and above	22 (58%)
Gender	
Female	24 (63%)
Male	14 (37%)
Marital status	
Single	5 (13%)
Married	39 (76%)
Divorced/separated	1 (3%)
Widowed	3 (8%)
Education (highest level completed)	
No formal education	9 (23%)
Primary school or lower	7 (18%)
Middle school	12 (32%)
High school	4 (11%)
University	6 (16%)
Occupation	
In employment	21 (37%)
Not in employment	14 (55%)
Retired	3 (8%)
Relationship with person with schizophrenia	
Parent	17 (44%)
Sibling	5 (13%)
Child	4 (11%)
Spouse	8 (21%)
Family by marriage	4 (11%)

**Table 2:** Relative importance of outcomes for PwS and primary caregivers.

Outcome domains (Pw*S* = 32)	Outcome domains (Primary caregiver*s* = 38)
Symptom control	Activity
(*f* = 17, *s* = 37)	(*f* = 25, *s* = 53)
Employment/education	Employment/education
(*f* = 14, *s* = 28)	(*f* = 22, *s* = 51)
Social functioning	Fulfilment of duties and responsibilities
(*f* = 14, s = 16)	(*f* = 23, *s* = 47)
Activity	Social functioning
(*f* = 8, *s* = 16)	(*f* = 21, *s* = 45)
Management without medication	Symptom control
(*f* = 8, *s* = 14)	(*f* = 23, *s* = 35)
Cognitive ability	Independent functioning
(*f* = 8, *s* = 12)	(*f* = 20, *s* = 35)
Reduced side-effects	Self-care
(*f* = 8, *s* = 9)	(*f* = 18, *s* = 26)
Fulfilment of duties and responsibilities	Self-determination
(*f* = 7, *s* = 9)	(*f* = 17, *s* = 33)
Self-care	Reduced side-effects
(*f* = 6, *s* = 9)	(*f* = 6, *s* = 9)
Independent functioning	Management without medication
(*f* = 5, *s* = 7)	(*f* = 5, *s* = 7)
Self-determination	Cognitive ability
(*f* = 5, *s* = 7)	(*f* = 4, *s* = 5)

PwS: Persons with schizophrenia; *f*: Frequency of the outcome; *s*: Saliency of the outcome.

**Table 3:** Domains of recovery from schizophrenia in India.

Domains of clinical recovery	Domains of functional recovery
Symptom control	Employment/education
Reduced side-effects	Social functioning
Cognitive ability	Activity
	Independent functioning
	Fulfilment of duties and responsibilities
	Self-care
	Self-determination
	Management without medication
